# Développement et validation de la version canadienne-française de l’échelle de Satisfaction des Adolescents de la gestion de la Douleur postopératoire – Scoliose idiopathique (SAD-S)

**DOI:** 10.1080/24740527.2017.1324947

**Published:** 2017-07-27

**Authors:** Ariane Ballard, Sylvie Le May, Christelle Khadra, Jean Théroux, Sylvie Charette, Edith Villeneuve, Jill Chorney, Christophe Fortin, Stefan Parent

**Affiliations:** a Université de Montréal, Québec, Canada; b Centre de recherche du CHU Sainte-Justine, Montréal, Québec, Canada; c Centre Universitaire de Santé McGill, Montréal, Québec, Canada; d Faculté de Médecine, Université Dalhousie, Halifax, Nouvelle-Écosse, Canada; e IWK Health Centre, Halifax, Nouvelle-Écosse, Canada; f Cégep régional de Lanaudière, Terrebonne, Québec, Canada

**Keywords:** adolescents, douleur post-opératoire, instrument de mesure, pain management, satisfaction, scale development and validation, scoliose idiopathique, scoliosis, spinal fusion, validation d’instrument

## Abstract

**Background**: Spinal fusion for scoliosis generates moderate to severe pain intensity. There are currently no instruments available to measure adolescents’ satisfaction regarding post-spinal fusion pain management.

**Aims**: To develop and validate a scale on satisfaction of adolescents regarding pain management following spinal fusion for scoliosis.

**Methods**: A methodological design was used to develop and validate the French-Canadian scale “Satisfaction des Adolescents de la gestion de la Douleur postopératoire – Scoliose idiopathique (SAD-S)”. A modified Delphi method, with seven healthcare professionals and 10 adolescents, was used to establish content validity of the SAD-S. A pre-test of the scale was conducted with 10 adolescents post-spinal fusion. The final version of the scale was validated through a pilot study with 98 adolescents following their surgery.

**Results**: The SAD-S scale includes a total of 13 items. Principal component analysis yielded a two-factor structure (2 subscales): 1) Pain management education and 2) Education regarding medication. These two factors explained 47,8% of the total variance for satisfaction. A Cronbach’s alpha of 0,84 was obtained for internal consistency.

**Conclusion**: Validation of the SAD-S scale showed that it has good psychometric properties with this population. Further validation is required with a larger sample to pursue its validation.

## RéSUMé

**Introduction**: La chirurgie correctrice de la scoliose génère de la douleur postopératoire d’intensité modérée à sévère. Il n’existe pas d’instruments sur la satisfaction d’adolescents ayant eu recours à ce type de chirurgie quant à la gestion de leur douleur.

**Objectifs**: Développement et validation d’une échelle pour mesurer la satisfaction d’adolescents ayant subi une chirurgie correctrice de la scoliose quant à la gestion de leur douleur postopératoire.

**Méthodes**: Un devis méthodologique a été utilisé pour développer l’Échelle de Satisfaction des Adolescents de la gestion de la Douleur postopératoire – Scoliose idiopathique (SAD-S). La validité de contenu du SAD-S a été évaluée selon la méthode Delphi modifiée avec sept professionnels de la santé et 10 adolescents. Un pré-test de l’instrument a été effectué auprès de 10 autres adolescents ayant subi cette chirurgie. Une étude de validation a ensuite été menée auprès de 98 adolescents en phase postopératoire.

**Résultats**: Le SAD-S comprend 13 énoncés. L’analyse en composantes principales a permis de déterminer une structure à deux facteurs (sous-échelles): 1) enseignement sur la gestion de la douleur et 2) enseignement sur la médication. Ces facteurs permettent d’expliquer 47,8 % de la variance totale pour la satisfaction. Un alpha de Cronbach de 0,84 a été obtenu pour la consistance interne de l’échelle.

**Conclusion**: Le SAD-S possède de bonnes propriétés psychométriques pour la mesure de la satisfaction auprès de cette clientèle. La validation de cet instrument devra être poursuivie à l’aide d’un échantillon plus grand.

## Introduction

La scoliose idiopathique est une déformation tridimensionnelle de la colonne vertébrale d’origine inconnue qui afflige 2 à 3 % des adolescents.^1,2^ La mesure de l’angle de Cobb permet de déterminer le degré de courbure de la colonne vertébrale. On diagnostique une scoliose idiopathique lorsque l’angle de Cobb est ≥ 10°.^1^ Près de 0,3 % des adolescents ayant une scoliose idiopathique présentent une courbure majeure et évolutive de plus de 40° nécessitant une chirurgie correctrice de la scoliose ayant pour objectif de stabiliser la difformité, tout en prévenant la progression de la déviation.^2-4^ Cette chirurgie, qui nécessite une hospitalisation d’environ 7 à 8 jours, génère des niveaux d’intensité de douleur postopératoire de modérés à sévères.^5-7^

Une revue exhaustive de la littérature n’a pu recenser d’études ayant évalué la satisfaction en lien avec la gestion de la douleur postopératoire lors d’une chirurgie correctrice de la scoliose. Les études recensées portaient davantage sur la satisfaction postopératoire en général^8-10^ ou bien sur la qualité de vie postopératoire.^11-13^ D’ailleurs, certaines études portant sur les perceptions des adolescents suite à une chirurgie correctrice de la scoliose associaient davantage la satisfaction au succès de la chirurgie, c’est-à-dire à la correction de la difformité et à l’aspect esthétique,^14.15^ qu’à la gestion de la douleur. L’instrument de mesure le plus fréquemment utilisé dans les études reliées à la satisfaction (qualité de vie) post-opératoire lors d’une chirurgie correctrice de la scoliose est le *Scoliosis Research Society*-30 (SRS-30) ou l’un de ses dérivés.^4,16,17^ Cependant, cet instrument permet d’abord et avant tout de mesurer la qualité de vie suite à la chirurgie plutôt que la satisfaction et comporte seulement quatre questions générales sur la gestion de la douleur.^17-19^ La revue de la littérature a également permis de recenser un instrument permettant d’évaluer la satisfaction des patients ayant une douleur aiguë ou chronique, soit le *Pain Treatment Satisfaction Scale* (PTSS).^20^ Bien que le PTSS possède d’excellentes propriétés psychométriques et qu’il ait été développé selon un processus rigoureux, il évalue la satisfaction de la gestion de la douleur en général et n’est pas spécifique à aucune procédure ou population particulière. Ainsi, puisque la chirurgie correctrice de la scoliose génère des niveaux de douleur importants^5-7^ et que ce sont les adolescents qui représentent la population cible de cette chirurgie,^1,2^ il est nécessaire d’avoir recours à un instrument de mesure adapté et davantage personnalisé.

L’évaluation de la satisfaction de patients quant à la gestion de leur douleur est un aspect non négligeable du processus chirurgical. Avec la popularité des approches centrées sur le patient, la recension des facteurs jouant un rôle dans la satisfaction de celui-ci quant à la gestion de sa douleur s’avère essentielle. Toutefois, à ce jour, nous n’avons pu identifier d’instrument spécifique permettant de mesurer la satisfaction des adolescents suite à une chirurgie correctrice de la scoliose. Le but de cette étude était de développer et de valider un instrument de mesure permettant de mesurer la satisfaction d’adolescents quant à la gestion de leur douleur postopératoire suite à une chirurgie correctrice de la scoliose.

## Méthode

### Devis

Tel que suggéré par Streiner et Norman,^21^ un devis méthodologique a été utilisé pour procéder au développement et à la validation de la version canadienne-française de l’Échelle de Satisfaction des Adolescents quant à la gestion de la Douleur postopératoire - Scoliose idiopathique (SAD-S).

#### Développement de l’instrument de mesure

**Figure 1.**
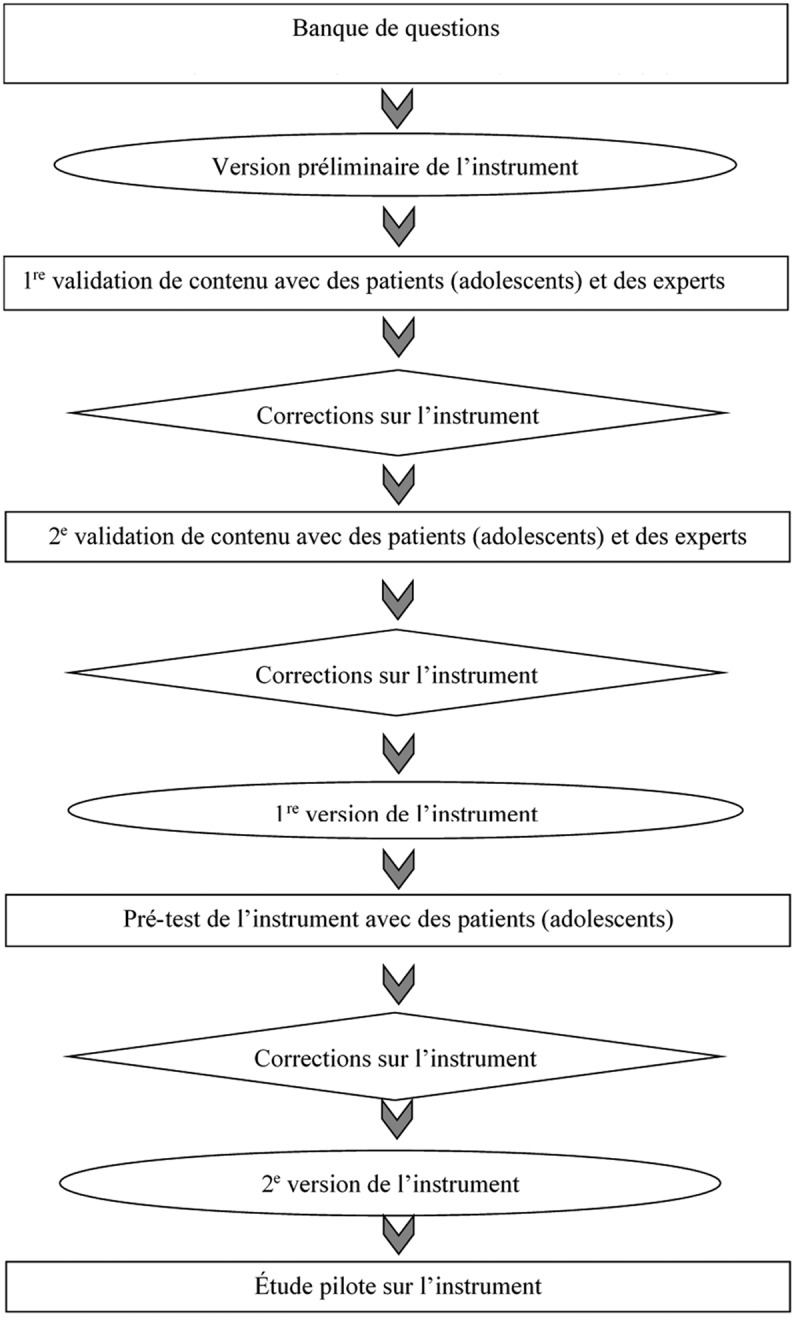
Principales étapes du développement d’un instrument de mesure. Inspirées de Le May et al. (2001).^22^ Les rectangles représentent les différentes phases de la validation de l’instrument. Les losanges représentent les modifications effectuées sur l’instrument. Les ellipses représentent les différentes versions de l’instrument.

Nous avons procédé de façon systématique pour le développement de l’instrument de mesure SAD-S, tel que proposé par Le May et al. (Figure 1).^22^

##### Génération des énoncés

Une revue exhaustive de la littérature a été menée dans diverses bases de données afin de déterminer les différents énoncés à inclure dans un instrument de mesure portant sur la satisfaction de la gestion de la douleur postopératoire. À cet effet, le SRS-30, le PTSS,^20^ le *Brief Pain Inventory*,^23^ l’*Adolescent Pediatric Pain Tool*^24^ et le *Treatment Outcomes of Pain Survey*^25^ comptent parmi les instruments de mesure qui ont été consultés pour procéder à la génération des énoncés. À partir des résultats de cette revue de la littérature et de l’expérience clinique des auteurs, une version préliminaire de l’instrument de mesure a été générée.^22^ Cette version comportait un total de 38 énoncés.

##### Validité de contenu et réduction des énoncés

La validité de contenu a été évaluée en soumettant la version préliminaire de l’instrument de mesure à un panel de sept professionnels de la santé aux compétences diversifiées (deux infirmières cliniciennes en gestion de la douleur et en chirurgie pédiatrique; deux infirmières en orthopédie pédiatrique; un chercheur en gestion de la douleur; un anesthésiologiste pédiatrique; et un chirurgien en orthopédie pédiatrique) et à cinq adolescents ayant subi une chirurgie correctrice de la scoliose. La méthode Delphi modifiée^26-28^ a été utilisée auprès des experts et des adolescents afin d’obtenir un consensus sur les différents énoncés de l’instrument quant à leur pertinence, leur clarté et leur importance. Ce processus a permis de déterminer les énoncés qui étaient moins pertinents et ceux qui manquaient de clarté (1^e^ validité de contenu). Cette version préliminaire du SAD-S a été soumise une seconde fois au même processus et auprès du même panel de professionnels de la santé, mais auprès de cinq nouveaux adolescents. Ce panel a été invité à modifier, ajouter ou supprimer les énoncés qu’il considérait encore ambigus (2^e^ validité de contenu). À l’issue de ces deux processus de validité de contenu, une première version officielle de l’instrument de mesure SAD-S a été établie. Ces processus ont permis de réduire le nombre d’énoncés de 38 à 13.

##### Pré-test

La première version de l’instrument a été soumise à un pré-test auprès de 10 adolescents ayant subi une chirurgie correctrice de la scoliose et n’ayant pas participé aux étapes précédentes de validité de contenu. Ce pré-test a permis de s’assurer que les différents énoncés de l’instrument de mesure étaient compréhensibles et dénués de toute ambigüité ainsi que d’apporter certains correctifs. Suite aux corrections proposées, cette version finale de l’échelle SAD-S a été soumise à une validation auprès d’un plus grand échantillon.

##### L’échelle SAD-S

Le SAD-S est une échelle d’évaluation de la satisfaction d’adolescents concernant la gestion de leur douleur postopératoire suite à une chirurgie correctrice de la scoliose idiopathique. Le SAD-S comprend un total de 13 énoncés regroupés en deux sous-échelles, soit: 1) Enseignement sur la gestion de la douleur (10 énoncés) et 2) Enseignement sur la médication (3 énoncés). Chacun de ces énoncés est évalué à l’aide d’une échelle de type Likert en six points allant de 1 (Peu satisfait) à 6 (Très satisfait). Un score total est obtenu par l’addition des scores de chacune des deux dimensions. Le score total minimal possible est de 13 et le score total maximal possible est de 78. Un score élevé signifie une satisfaction élevée quant à la gestion de la douleur postopératoire suite à une chirurgie correctrice de la scoliose. L’échelle SAD-S s’accompagne également d’une section portant sur l’importance des énoncés de satisfaction ainsi que d’une section permettant de collecter des données cliniques, telles que l’intensité de la douleur, les effets secondaires et le format de la médication. Ces données sont complémentaires à l’échelle SAD-S.

### Validation de l’instrument de mesure

Afin de procéder à la validation de l’instrument de mesure SAD-S, nous l’avons soumis à des adolescents en phase postopératoire d’une chirurgie correctrice de la scoliose. Selon les recommandations de Tinsley et Tinsley,^29^ un ratio de 5 à 10 participants par énoncé est adéquat pour mener une analyse factorielle. Ainsi, il a été planifié de recruter entre 65 et 130 participants.

#### Milieu et participants

Le recrutement des participants a eu lieu de septembre 2012 à juin 2014. Les participants ont été recrutés au département de chirurgie du CHU Sainte-Justine, qui est un centre hospitalier universitaire pédiatrique de la région de Montréal. Les critères d’inclusion étaient les suivants: 1) être âgé de 10 à 20 ans; 2) avoir subi une chirurgie de type arthrodèse postérieure ou antérieure de première intention ou une combinaison des deux procédures; 3) être en mesure de s’exprimer et d’écrire en français. Les adolescents présentant un diagnostic de déficit cognitif modéré ou de retard mental sévère ou ayant eu une convalescence en centre de réadaptation ont été exclus. L’étude a été approuvée par le Comité d’éthique et de la recherche du CHU Sainte-Justine (Montréal, Canada). Un formulaire de consentement a été signé par les parents et l’assentiment des adolescents a été obtenu préalablement à leur participation à l’étude.

#### Recrutement des participants

Les participants admissibles à l’étude ont été approchés lors du 7^e^ jour postopératoire avant leur congé hospitalier. Ils ont été invités à compléter le SAD-S au 10^e^ jour suivant leur congé du centre hospitalier. Ce temps de mesure était justifié par le fait qu’il est préférable d’évaluer la satisfaction des soins reçus seulement lorsque le patient a quitté l’hôpital, et ce, afin d’éviter un biais relié à la désirabilité sociale ou bien un biais d’évaluation positif relié à la crainte des participants de subir des préjudices en cas d’une évaluation négative des services.

#### Analyses psychométriques et statistiques

La validité de construit a été évaluée à l’aide d’une analyse en composantes principales (ACP) avec rotation orthogonale Varimax [30], puisqu’elle permet de déterminer une solution où les composantes sont indépendantes tout en maximisant la variance expliquée.^31^ La mesure de Keyser-Meyer-Olkin (KMO) et le test de sphéricité de Bartlett ont été menés préalablement à l’ACP. Plusieurs critères de dimensionnalité ont permis d’établir le nombre de facteurs à extraire, soit le critère de Kaiser-Guttman (*Eigenvalue rule*),^32^ le test du coude de Cattell (*Scree test*)^33^ ainsi que l’analyse parallèle de Horn.^34^ Différentes analyses de consistance interne ont été utilisées pour mesurer la fidélité de l’instrument, telles que les corrélations inter-énoncés, les corrélations énoncés-total^21^ ainsi que le coefficient alpha de Cronbach.^35^ Des statistiques descriptives ont été utilisées pour décrire les caractéristiques des participants à l’étude ainsi que pour rapporter les données cliniques. Les analyses statistiques ont été menées avec le logiciel SPSS version 24 (IBM Corporation, USA).

## Résultats

### Caractéristiques des participants

Parmi les 113 adolescents ayant été approchés pour participer à l’étude, un total de 98 (87,0%) a retourné le questionnaire complété. Parmi ceux-ci, trois ont dû être exclus de l’analyse des résultats en raison de données manquantes (95/98). La majorité des participants étaient des filles (89,8 %) et l’âge moyen des participants était de 16.3 ± 1.6 ans.

#### Qualités psychométriques de l’échelle SAD-S

##### Validité de construit

**Figure 2.**
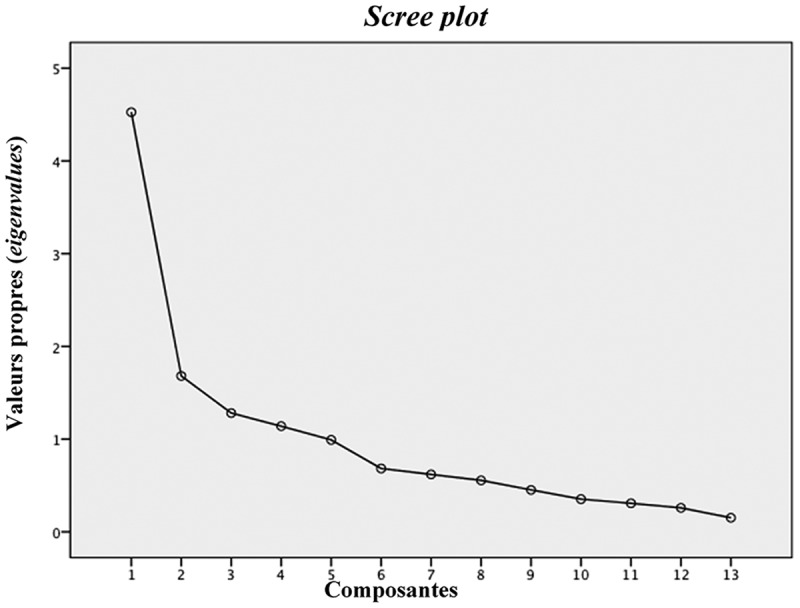
Test du coude de Cattell (*scree plot*)^33^ représenté par le graphique des valeurs propres (*eigenvalues*).

**Figure 3.**
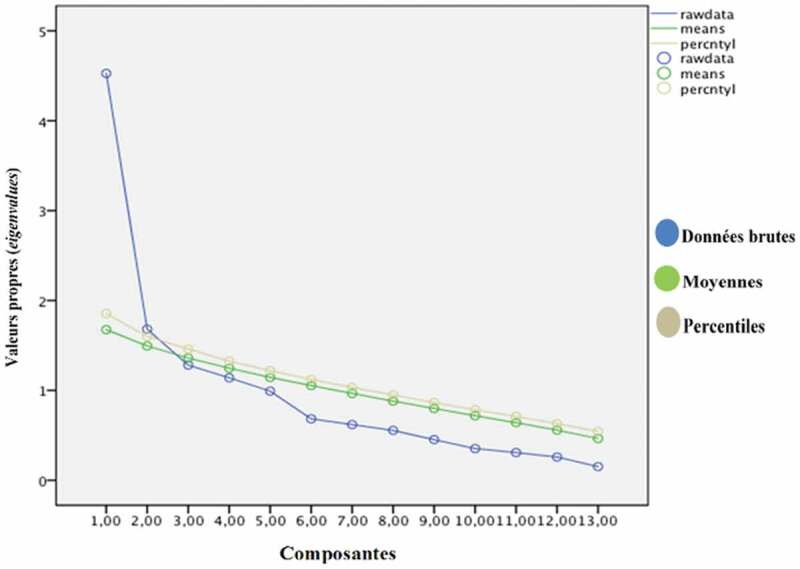
Résultats de l’Analyse paralléle de Horn.^34^

La mesure de KMO (0,738) et le test de sphéricité de Bartlett (p < 0,000) ont indiqué qu’il était approprié de mener une analyse factorielle en composantes principales avec rotation orthogonale Varimax.^31^ En ce qui concerne le nombre de facteurs à extraire, le critère de Kaiser-Guttman^32^ a indiqué la présence de quatre facteurs significatifs ayant une valeur propre initiale supérieure à 1. Le test du coude de Cattell (Figure 2),^33^ qui est représenté par le graphique des valeurs propres, a plutôt révélé qu’une structure à trois facteurs permettait de maximiser la variance expliquée. Toutefois, l’analyse parallèle de Horn^34^ a quant à elle permis de répertorier seulement deux facteurs (Figure 3). Puisqu’il s’agit de la technique qui est la plus fiable et recommandée pour déterminer le nombre de facteurs à extraire lors d’une ACP,^31,36-40^ une solution à deux facteurs a donc été privilégiée. Le Tableau 1 présente la matrice des composantes en rotation orthogonale Varimax à deux facteurs. Le premier facteur, avec une valeur propre de 4,5, a été nommé « Enseignement sur la gestion de la douleur » (10 énoncés) et permet d’expliquer à lui seul 34,8 % de la variance totale. Le second facteur, soit « Enseignement sur la médication » (3 énoncés) représente 12,9 % de la variance totale avec une valeur propre de 1,7. Ces deux facteurs permettent d’expliquer 47,8 % de la variance totale des 13 énoncés de l’échelle de mesure SAD-S (Tableau 2).

**Tableau 1.** Analyse en composantes principales avec rotation orthogonale Varimax selon une solution à deux facteurs (n=92).

Énoncés	Facteurs	
	1	2
Énoncé 1: L’intensité de la douleur	**0,615**	0,170
Énoncé 2: La médication utilisée pour diminuer la douleur	**0,539**	0,417
Énoncé 3: La façon d’utiliser l’échelle pour mesurer la douleur	**0,431**	0,413
Énoncé 4: Les effets secondaires que tu pourrais avoir	**0,479**	0,308
Énoncé 5: Les médicaments utilisés au retour à la maison et leurs effets secondaires	**0,514**	0,368
Énoncé 6: Te croire quand tu leurs parles de ta douleur	**0,724**	-0,254
Énoncé 7: T’aider à trouver une position confortable dans ton lit pour diminuer la douleur	**0,584**	0,098
Énoncé 8: Te poser des questions par rapport à la douleur que tu ressens quand tu respires profondément, t’assoies ou te déplaces.	**0,517**	0,295
Énoncé 9: Te demander ton niveau de douleur, sur une échelle de 1 à 10, à tous les matins, après-midis et soirées.	**0,474**	0,130
Énoncé 10: Traiter ta douleur jusqu’à ce qu’elle soit soulagée	**0,673**	0,165
Énoncé 11: Le temps que prend la médication avant de soulager ta douleur	0,008	**0,855**
Énoncé 12: Le niveau de soulagement de la douleur que t’apporte ta médication	0,199	**0,874**
Énoncé 13: La durée du soulagement de la douleur que t’apporte ta médication	0,238	**0,807**

Facteurs: 1-Enseignement sur la gestion de la douleur, 2-Enseignement sur la médication

**Tableau 2.** Valeurs propres initiales (*eigenvalues*) et variance totale expliquée.

Facteurs	Valeurs propres initiales	% de la variance totale	% de la variance cumulée
Facteur 1	4,526	34,814	34,814
Facteur 2	1,681	12,932	47,746
Facteur 3	1,281	9,854	57,600
Facteur 4	1,140	8,767	66,368

##### Fidélité

Un coefficient alpha de Cronbach de 0,84 a été obtenu pour l’ensemble de l’échelle composée des 13 énoncés. En ce qui concerne la cohérence interne des deux sous-échelles, des alphas de Cronbach de 0,80 et 0,86 ont été calculés respectivement pour les sous-échelles « Enseignement sur la gestion de la douleur » et « Enseignement sur la médication ». L’analyse de la matrice de corrélations inter-énoncés a permis de constater que la majorité des corrélations entre les 13 énoncés sont supérieures à 0,30 et inférieures à 0,70 (Tableau 3). Le calcul des corrélations entre chacun des énoncés et l’échelle totale (énoncés-total) a également permis de déterminer que chacun des énoncés permettait d’assurer l’homogénéité de l’échelle de mesure SAD-S (Tableau 4). Le Tableau 4 permet également de constater que si l’on retirait l’un des 13 énoncés de l’instrument de mesure, l’alpha de Cronbach se verrait diminué, ce qui signifie que la consistance interne de l’échelle est maximisée en présence des 13 énoncés.

**Tableau 3.** Matrice de corrélations inter-items.

	Énoncé 1	Énoncé 2	Énoncé 3	Énoncé 4	Énoncé 5	Énoncé 6	Énoncé 7	Énoncé 8	Énoncé 9	Énoncé 10	Énoncé 11	Énoncé 12	Énoncé13
Énoncé 1	1,000	0,445	0,191	0,304	0,436	0,233	0,106	0,411	0,289	0,373	0,153	0,175	0,283
Énoncé 2	0,445	1.000	0,396	0,354	0,537	0,238	0,232	0,272	0,257	0,267	0,337	0,368	0,373
Énoncé 3	0,191	0,396	1.000	0,258	0,259	0,293	0,199	0,273	0,299	0,275	0,340	0,364	0,402
Énoncé 4	0,304	0,354	0,258	1.000	0,587	0,209	0,258	0,102	0,094	0,270	0,175	0,336	0,317
Énoncé 5	0,436	0,537	0,259	0,587	1.000	0,120	0,255	0,287	0,136	0,199	0,200	0,351	0,313
Énoncé 6	0,233	0,238	0,293	0,209	0,120	1.000	0,363	0,092	0,366	0,386	-0,109	0,037	0,059
Énoncé 7	0,106	0,232	0,199	0,258	0,255	0,363	1.000	0,394	0,020	0,489	0,094	0,273	0,275
Énoncé 8	0,411	0,272	0,273	0,102	0,287	0,092	0,394	1.000	0,329	0,420	0,280	0,299	0,305
Énoncé 9	0,289	0,257	0,299	0,094	0,136	0,366	0,020	0,329	1.000	0,217	0,158	0,276	0,186
Énoncé 10	0,372	0,267	0,275	0,270	0,199	0,386	0,489	0,420	0,217	1.000	0,219	0,326	0,317
Énoncé 11	0,153	0,337	0,340	0,175	0,200	-0,109	0,094	0,280	0,158	0,219	1.000	0,707	0,559
Énoncé 12	0,175	0,368	0,364	0,336	0,351	0,037	0,273	0,299	0,276	0,326	0,707	1.000	0,772
Énoncé 13	0,283	0,373	0,402	0,317	0,313	0,059	0,275	0,305	0,182	0,317	0,559	0,772	1.000

**Tableau 4.** Consistance interne.

Énoncés (k)	Alpha de Cronbach si suppression d’un item (k-1)	Corrélation entre chacun des items et l’échelle totale
1. L’intensité de la douleur	0,828	0,482
2. La médication utilisée pour réduire la douleur	0,820	0,592
3. La façon d’utiliser l’échelle pour mesurer la douleur	0,827	0,500
4. Les effets secondaires que tu pourrais avoir	0,829	0,470
5. Les médicaments utilisés au retour à la maison et leurs effets secondaires	0,824	0,537
6. Te croire lorsque tu leur parles de ta douleur	0,838	0,317
7. T’aider à trouver une position confortable dans ton lit pour diminuer la douleur	0,833	0,412
8. Te poser des questions à propos de la douleur que tu ressens quand tu respires profondément, t’assoies ou te déplaces	0,828	0,485
9. Te demander ton niveau de douleur, sur une échelle de 1 à 10, à tous les matins, après-midis et soirées	0,835	0,360
10. Ils traitent ta douleur jusqu’à ce que tu sois soulagé	0,825	0,532
11. Le temps que prend la médication avant de soulager ta douleur	0,831	0,438
12. Le niveau de soulagement de la douleur que t’apporte ta médication	0,819	0,617
13. La durée du soulagement de la douleur que t’apporte ta médication	0,820	0,596

#### Données cliniques

##### Échelle SAD-S

Les résultats de l’échelle SAD-S ont démontré qu’en moyenne, les participants ont été très satisfaits quant à la gestion de leur douleur postopératoire (62.2 ± 8.3 sur 78.0). De manière plus précise, des scores de satisfaction élevés ont été enregistrés pour les deux sous-échelles. En effet, les participants ont rapporté un score moyen de 4,8 ± 1,0 sur 6 pour la sous-échelle « Enseignement sur la gestion de la douleur » et un score moyen de 4,6 ± 1,0 sur 6 pour la sous-échelle « Enseignement sur la médication ». Les énoncés dont les participants ont été le plus satisfaits sont « Te poser des questions à propos de la douleur que tu ressens quand tu respires profondément » (5,4 ± 1,0 sur 6), « Traiter ta douleur jusqu’à ce qu’elle soit soulagée » (5,2 ± 1,0 sur 6) et « T’aider à trouver une position confortable dans ton lit pour diminuer la douleur » (5,1 ± 1,1 sur 6). Les énoncés dont les participants ont été le moins satisfaits concernent la durée que prend la médication actuelle avant de soulager la douleur (4,4 ± 1,0 sur 6) ainsi que l’information reçue après l’opération par rapport aux effets secondaires (4,3 ± 1,3 sur 6) et à l’intensité de la douleur (4,5 ± 1,1 sur 6). Le Tableau 5 présente les résultats sur la satisfaction des adolescents selon les deux sous-échelles.

**Tableau 5.** Satisfaction des adolescents pour chacune des sous-échelles et des items de l’échelle SAD-S (n = 92).

Sous-échelles	Score moyen global (Moyenne ± écart-type)
**Sous-échelle: Enseignement sur la gestion de la douleur**	
L’intensité de la douleur	4,5 ± 1,1 sur 6
La médication utilisée pour diminuer la douleur	4,9 ± 1,1 sur 6
La façon d’utiliser l’échelle pour mesure la douleur	4,6 ± 1,2 sur 6
Les effets secondaires que tu pourrais avoir	4,2 ± 1,3 sur 6
Les médicaments utilisés au retour à la maison et leurs effets secondaires	4,5 ± 1,2 sur 6
Te croire lorsque tu leur parles de ta douleur	5,2 ± 1,0 sur 6
T’aider à trouver une position confortable dans ton lit pour diminuer la douleur	5,1 ± 1,1 sur 6
Te poser des questions à propos de la douleur que tu ressens quand tu respires profondément, t’assoies ou te déplaces	5,0 ± 1.0 sur 6
Te demander ton niveau de douleur, sur une échelle de 1 à 10, à tous les matins, après-midis et soirées	5,3 ± 1,0 sur 6
Traiter ta douleur jusqu’à ce qu’elle soit soulagée	5,2 ± 1,0 sur 6
**Score moyen**	4,8 ± 1,0 sur 6
**Sous-échelle: Enseignement sur la médication**	
La temps que prend la médication avant de soulager ta douleur	4,4 ± 1,0 sur 6
Le niveau de soulagement de la douleur que t’apporte ta médication	4,8 ± 1,0 sur 6
La durée du soulagement de la douleur que t’apporte ta médication	4,7 ± 1,1 sur 6
**Score moyen**	4,6 ± 1,0 sur 6
**Score total**	62,2 ± 8,3 sur 78

##### Importance des critères de satisfaction

Les participants ont en moyenne accordé beaucoup d’importance à la gestion de leur douleur postopératoire (5,01 ± 0,4 sur 6). Les énoncés auxquels les participants ont accordé le plus d’importance concernent les actions entreprises par les infirmières et les médecins, soit qu’ils les croient lorsqu’ils parlent de leur douleur (5,6 ± 0,7 sur 6), qu’ils les aident à trouver une position confortable dans leur lit (5,6 ± 0,9 sur 6) et qu’ils traitent leur douleur jusqu’à ce qu’elle soit soulagée (5.5 ± 0,8 sur 6).

##### Intensité de la douleur

Environ 10 jours après leur congé du centre hospitalier, les participants ont rapporté, en moyenne, une intensité de la douleur légère (2,6 ± 1,9). Concernant la plus importante douleur ressentie au cours de la dernière semaine, les participants ont rapporté, en moyenne, une douleur d’intensité modérée de 5,9 ± 2,2 sur une échelle de 0 à 10, Finalement, le niveau de douleur ressenti le plus souvent dans la dernière semaine par les participants était de 3,4 ± 1,9.

##### Effets secondaires et format de la médication

Les effets secondaires qui ont le plus dérangé les participants étaient les hallucinations (4,1 ± 2,3 sur 6,0) et les sensations bizarres non désagréables (3,94 ± 1,98). La constipation et les douleurs abdominales (3,3 ± 1,8 sur 6,0) sont, quant à eux, les effets secondaires qui ont le moins dérangé les participants. Pour ce qui est de la satisfaction du format de la médication, les participants ont rapporté un score de satisfaction moyen de 4,5 ± 1,9 sur un total de 6,0. De manière plus précise, ils ont été le plus satisfait de la pompe d’auto-analgésie 5,2 ±1,0 sur 6,0 et ont été le moins satisfait de la médication intra-rectale 3,6 ± 1,5 sur 6,0.

## Discussion

Cette étude a permis de développer la toute première échelle de mesure permettant d’évaluer la satisfaction de la gestion de la douleur postopératoire d’adolescents ayant eu recours à une chirurgie correctrice de la scoliose. Cette dernière a été développée selon un processus rigoureux, ce qui a permis de s’assurer que les énoncés inclus reflètent les besoins identifiés quant à la population à laquelle il est destiné. L’échelle SAD-S comporte 13 énoncés regroupés selon deux dimensions relatives à l’enseignement reçu sur la gestion de la douleur et sur la médication. L’échelle SAD-S semble avoir été bien acceptée par les participants puisque la grande majorité d’entre eux ont répondu aux 13 énoncés (> 95%). Cette étude de validation a permis d’établir que le SAD-S possède des données psychométriques adéquates pour cette population.

En ce qui concerne la validité de construit, l’ACP a permis de mettre en évidence une structure à deux facteurs. Tel que suggéré par Conway et Huffcut,^41^ plusieurs méthodes ont été utilisées pour déterminer le nombre de facteurs à extraire, ce qui a permis d’augmenter la rigueur de la démarche. Ainsi, même si le critère Kaiser-Guttman^32^ et le test du coude de Cattell^33^ suggéraient respectivement la présence de quatre et trois facteurs significatifs, l’analyse parallèle de Horn^34^ a plutôt déterminé une solution à deux facteurs. Bien que le critère de Kaiser-Guttman^32^ soit certainement le plus connu et le plus utilisé en pratique, il est largement critiqué pour ne pas tenir compte de l’erreur d’échantillonnage^36^ ainsi que pour sa tendance à surestimer le nombre de facteurs.^34,41^ La fiabilité du test du coude de Cattell^33^ est également remise en question en raison de sa subjectivité.^42,43^ En effet, étant donné qu’il n’y a pas de définition objective du point de rupture (coude) permettant de distinguer les facteurs significatifs des facteurs non significatifs, l’interprétation du graphique peut être ambiguë et variable.^42,43^ Considérant les limites du critère de Kaiser-Guttman et du test du coude de Cattell, ces derniers ne sont plus considérés comme étant des méthodes de choix pour déterminer le nombre de facteurs à extraire lors d’une ACP.^37^ Enfin, l’analyse parallèle de Horn^34^ permet de pallier les limites des deux méthodes en générant de manière aléatoire différentes matrices de données en parallèle avec la matrice de corrélations générée par l’ACP. De manière plus précise, chacune des matrices de données aléatoires compte le même nombre de participants et de variables que la matrice de corrélations.^42,43^ Un facteur est donc considéré comme étant significatif lorsque sa valeur propre est plus grande que la moyenne des valeurs propres des matrices de données aléatoires.^36,42^ Parmi les nombreuses méthodes disponibles pour déterminer le nombre de facteurs à extraire, l’analyse parallèle de Horn^34^ est sans contredit la plus recommandée dans la littérature^31,36-40^ puisqu’elle prend en considération l’erreur d’échantillonnage [36] et permet de minimiser la sensibilité à différents facteurs.^37^ C’est donc la précision et la rigueur de cette méthode qui ont permis d’appuyer le choix d’une structure à deux facteurs. De plus, c’est cette structure qui permettait d’obtenir les meilleurs coefficients alpha de Cronbach pour chacune des sous-échelles.

Toutefois, la matrice des composantes en rotation orthogonale à deux facteurs indique que différents énoncés (n° 2, n° 3, n° 4, n° 5) présentent une saturation factorielle > 0,40 sur plus d’un facteur, ce qui laisse suggérer qu’ils pourraient être associés à la fois aux facteurs 1 et 2. Il a tout de même été décidé de conserver ces énoncés, puisque la suppression de l’un d’eux entrainait une diminution du coefficient alpha de Cronbach de l’échelle et qu’ils étaient jugés comme étant suffisamment importants par les participants. Le recours à une solution à deux facteurs (deux sous-échelles) permet de cerner de manière plus précise les dimensions de la satisfaction à améliorer selon les participants. Enfin, les résultats confirment que l’instrument de mesure permet de bien mesurer le construit, soit la satisfaction des adolescents quant à la gestion de leur douleur postopératoire.

Le calcul des coefficients alpha de Cronbach et des corrélations inter-énoncés et énoncés-total ont permis d’établir la fidélité de l’échelle SAD-S comme étant satisfaisante. Les coefficients alpha de Cronbach de l’échelle SAD-S (0,84) et de ses sous-échelles (0,86; 0,80) indiquent une forte corrélation entre les énoncés et suggèrent que chacun d’eux mesure le même construit. En effet, le coefficient alpha de Cronbach devrait être supérieur à 0,80 pour être en mesure de conclure à la fidélité d’un instrument.^21,44^ Le calcul des corrélations inter-énoncés et énoncés-total permet de conclure à l’homogénéité de l’instrument de mesure puisque la majorité de celles-ci étaient dans les limites du seuil de discrimination admis, soit supérieures à 0,30, mais inférieures à 0,70.^45,46^ Ceci permet de statuer que chacun des énoncés évalue un attribut distinct du construit de l’échelle de mesure.^21^ Les analyses de fidélité ont également indiqué que la consistance interne du SAD-S était maximisée en présence des 13 énoncés.

D’autre part, pour conclure à la présence d’effets « plancher » et « plafond », il est nécessaire que plus de 15 % des participants aient obtenu le score maximal (max: 78) ou minimal (min: 13) possible sur l’échelle de mesure.^47,48^ Puisqu’aucun des participants n’a atteint l’un de ces scores, on considère qu’il y a une absence d’effets « plancher » et « plafond ». Ceci se traduit donc par une validité de contenu et une sensibilité au changement adéquates.^48^

En ce qui concerne les limites de l’étude, il est à noter que seule la validité de construit et la cohérence interne de l’échelle SAD-S ont été évaluées. Ainsi, dans une prochaine étude de validation, il pourrait être pertinent d’évaluer la fidélité à l’aide du test-retest et de la technique *split-half*. Il serait également pertinent d’évaluer les validités de critère et convergente du SAD-S en l’utilisant de manière concomitante avec une échelle évaluant le même construit, telle que la PTSS.^20^ Ainsi, bien que les propriétés psychométriques de l’échelle SAD-S laissent suggérer qu’il est un instrument de mesure valide auprès de cette population, il serait nécessaire de poursuivre sa validation de manière plus approfondie. La taille de l’échantillon constitue également une limite de cette étude. Bien que Tinsley et Tinsley^29^ suggèrent qu’un ratio de 5 à 10 participants par énoncé soit suffisant pour mener une analyse factorielle, plusieurs auteurs suggèrent un échantillon de plus grande envergure.^49-51^ Pour sa part, Comrey^51^ suggère qu’un échantillon de 200 participants est adéquat pour mener une analyse factorielle avec 40 énoncés et moins, alors que Rouquette et al.^49^ estiment que 300 participants sont nécessaires. Toutefois, considérant le nombre de chirurgies correctrices de la scoliose effectuées dans le centre hospitalier où s’est déroulée l’étude, il n’était pas réaliste de recruter autant de participants. Enfin, le développement et la validation de cette échelle ont été effectués dans un seul milieu, ce qui peut limiter la portée des résultats cliniques puisque les approches chirurgicales et plans de traitement de la douleur peuvent varier d’un centre hospitalier à un autre.

Il est à noter qu’une version anglaise de l’échelle SAD-S, soit la *Satisfaction of Adolescents with Postoperative Pain Management – Idiopathic Scoliosis* (SAP-S) a également été validée auprès d’adolescents âgés entre 10 et 18 ans ayant subi une chirurgie correctrice de la scoliose.^52^ Enfin, l’utilisation de cet instrument a permis d’obtenir des données cliniques quant aux soins associés à la gestion de la douleur postopératoire d’adolescents ayant subi une chirurgie correctrice de la scoliose. Ainsi, grâce à ses deux sous-échelles le SAD-S permettra de cibler de manière plus précise les besoins des patients en matière d’enseignement et de soulagement de la douleur postopératoire et ce, afin de développer des interventions adaptées.

## Conclusion

Cette étude a permis d’établir que le SAD-S possède une validité de construit et une fidélité adéquates pour la population concernée. L’utilisation de cette échelle de mesure représente une avenue intéressante pour identifier les éléments permettant d’assurer une satisfaction optimale quant à la gestion de la douleur postopératoire d’adolescents ayant subi une chirurgie correctrice de la scoliose. De plus, l’échelle SAD-S et les données cliniques pourraient permettre de guider le développement et l’identification d’interventions pharmacologiques et non-pharmacologiques permettant d’assurer et d’améliorer les pratiques relatives au soulagement de la douleur post-opératoire. La poursuite de la validation de cet outil avec un échantillon plus grand serait nécessaire afin d’obtenir davantage de données sur ses propriétés psychométriques.

## Remerciements

Les auteurs tiennent à remercier tous les patients et leur famille qui ont participé à l’étude ainsi que les cliniciens du milieu qui ont collaboré à la validité de contenu de l’échelle. Les auteurs (AB, SLM, CK, JT et JMC) sont membres du *Pain In Child Health (PICH)*, une initiative stratégique de formation en recherche sur la douleur pédiatrique des Instituts de recherche en santé du Canada.

## Supplementary Material

1324947_Supplemental_Material.docx
