# Supplementary material for: Développement et validation de la version canadienne-française de l’échelle de Satisfaction des Adolescents de la gestion de la Douleur postopératoire – Scoliose idiopathique (SAD-S)
Source: Can J Pain. 2017 Jul 27;1(1):50–60. doi: 10.1080/24740527.2017.1324947 (PMC8730624; doi:10.1080/24740527.2017.1324947)
Supplement: 1324947_Supplemental_Material.docx [file UCJP_A_1324947_SM5049.docx]

**Appendice**

***SAtisfaction de la gestion de la Douleur postopératoire - Scoliose idiopathique* (SAD-S)**

**Questionnaire pour les adolescents(e)**

© Le May, S.; Charette, S.; Dulude, N.; Lampron, A.; Joncas, J. (2013)

Code du patient :_______

***Le présent questionnaire vise à déterminer si les patients opérés pour scoliose idiopathique sont satisfaits de la gestion de la douleur au cours de la période ou ils sont à l’hôpital et au retour à la maison après l’opération. Le questionnaire ne vise pas à vérifier tes connaissances, car il n’y a pas de bonnes ou mauvaises réponses. Nous sommes seulement intéressées à connaître ton opinion et tes impressions. Nous te rappelons aussi que tes réponses ne seront pas mentionnées aux infirmières et aux médecins qui ont pris soin de toi quand tu étais à l’hôpital.***

***Nous te demandons de remplir le questionnaire le dixième jour après que tu sois retourné à la maison***

| **DATES:**  En premier lieu, nous aimerions connaître la date de ton opération et la date exacte à laquelle tu as rempli le questionnaire (même si tu n’as pas pu le remplir au 10^ème^ jour après ton retour à la maison). Cette information permettra de comparer tes réponses avec celles des autres patients. |
| --- |

**Date du congé** : **Date d’aujourd’hui**

___ / ___ / _______ ___ / ___ / _______

JJ / MM / AAAA JJ / MM / AAAA

**Échelle de mesure**

SAtisfaction de la gestion de la Douleur postopératoire – Scoliose idiopatique (SAD-S)

| **CONSIGNES :**  Les questions suivantes concernent les pratiques hospitalières par rapport à la gestion de la douleur postopératoire. Pour chaque énoncé, tu dois indiquer ton degré de satisfaction ou d’insatisfaction en **encerclant un chiffre** de **1 (peu satisfait)** à **6** **(très satisfait)**. Si l’énoncé ne s’applique pas, encercle N/A (non-applicable). |
| --- |

|  | **Peu satisfait(e)** | | **Satisfait(e)** | | **Très satisfait(e)** | | **N/A** |
| --- | --- | --- | --- | --- | --- | --- | --- |
| *Concernant* ***l’information*** *que tu as reçue* ***APRÈS*** *l’opération par rapport à :* |  |  |  |  |  |  |  |
| 1. L’intensité de la douleur | 1 | 2 | 3 | 4 | 5 | 6 | N/A |
| 2. La médication utilisée pour diminuer la douleur | 1 | 2 | 3 | 4 | 5 | 6 | N/A |
| 3. La façon d’utiliser l’échelle pour mesurer la douleur | 1 | 2 | 3 | 4 | 5 | 6 | N/A |
| 4. Les effets secondaires que tu pourrais avoir (ex. : mal au cœur, ça pique, tête qui tourne) | 1 | 2 | 3 | 4 | 5 | 6 | N/A |
| 5. Les médicaments utilisés au retour à la maison et leurs effets secondaires | 1 | 2 | 3 | 4 | 5 | 6 | N/A |
| *Concernant des* ***actions*** *posées par les* ***infirmières et les médecins*** |  |  |  |  |  |  |  |
| 6. Te croire quand tu leur parles de ta douleur | 1 | 2 | 3 | 4 | 5 | 6 | N/A |
| 7. T’aider à trouver une position confortable dans ton lit pour diminuer la douleur | 1 | 2 | 3 | 4 | 5 | 6 | N/A |
| 8. Te poser des questions par rapport à la douleur que tu ressens quand tu respires profondément, t’assoies ou te déplaces | 1 | 2 | 3 | 4 | 5 | 6 | N/A |
| 9. Te demander ton niveau de douleur, sur une échelle de 1 à 10, à tous les matins, après-midis et soirées | 1 | 2 | 3 | 4 | 5 | 6 | N/A |
| 10. Traiter ta douleur jusqu’à ce qu’elle soit soulagée | 1 | 2 | 3 | 4 | 5 | 6 | N/A |
| *Concernant ta médication* ***actuelle****:* |  |  |  |  |  |  |  |
| 11. La durée que prend la médication avant de soulager ta douleur | 1 | 2 | 3 | 4 | 5 | 6 | N/A |
| 12. La quantité de soulagement de la douleur que t’apporte ta médication | 1 | 2 | 3 | 4 | 5 | 6 | N/A |
| 13. La durée du soulagement de la douleur que t’apporte ta médication | 1 | 2 | 3 | 4 | 5 | 6 | N/A |

IMPORTANCE DES ÉNONCÉS DE L’ÉCHELLE DE MESURE SAD-S

| **CONSIGNES :**  Pour chaque énoncé, tu dois indiquer l’importance que tu lui accordes en **encerclant un chiffre** de **1 (peu important)** à **6** **(très important)**. Si l’énoncé ne s’applique pas, encercle N/A (non-applicable). |
| --- |

|  | **Peu important** | | **Important** | | **Très important** | | **N/A** |
| --- | --- | --- | --- | --- | --- | --- | --- |
| *Concernant* ***l’information*** *que tu as reçue* ***APRÈS*** *l’opération par rapport à :* |  |  |  |  |  |  |  |
| 1. L’intensité de la douleur | 1 | 2 | 3 | 4 | 5 | 6 | N/A |
| 2. La médication utilisée pour diminuer la douleur | 1 | 2 | 3 | 4 | 5 | 6 | N/A |
| 3. La façon d’utiliser l’échelle pour mesurer la douleur | 1 | 2 | 3 | 4 | 5 | 6 | N/A |
| 4. Les effets secondaires que tu pourrais avoir (ex. : mal au cœur, ça pique, tête qui tourne) | 1 | 2 | 3 | 4 | 5 | 6 | N/A |
| 5. Les médicaments utilisés au retour à la maison et leurs effets secondaires | 1 | 2 | 3 | 4 | 5 | 6 | N/A |
| *Concernant des* ***actions*** *posées par les* ***infirmières et les médecins*** |  |  |  |  |  |  |  |
| 6. Te croire quand tu leur parles de ta douleur | 1 | 2 | 3 | 4 | 5 | 6 | N/A |
| 7. T’aider à trouver une position confortable dans ton lit pour diminuer la douleur | 1 | 2 | 3 | 4 | 5 | 6 | N/A |
| 8. Te poser des questions par rapport à la douleur que tu ressens quand tu respires profondément, t’assoies ou te déplaces | 1 | 2 | 3 | 4 | 5 | 6 | N/A |
| 9. Te demander ton niveau de douleur, sur une échelle de 1 à 10, à tous les matins, après-midis et soirées | 1 | 2 | 3 | 4 | 5 | 6 | N/A |
| 10. Traiter ta douleur jusqu’à ce qu’elle soit soulagée | 1 | 2 | 3 | 4 | 5 | 6 | N/A |
| *Concernant ta médication* ***actuelle****:* |  |  |  |  |  |  |  |
| 11. La durée que prend la médication avant de soulager ta douleur | 1 | 2 | 3 | 4 | 5 | 6 | N/A |
| 12. La quantité de soulagement de la douleur que t’apporte ta médication | 1 | 2 | 3 | 4 | 5 | 6 | N/A |
| 13. La durée du soulagement de la douleur que t’apporte ta médication | 1 | 2 | 3 | 4 | 5 | 6 | N/A |

**Données cliniques**

EFFETS SECONDAIRES

| **CONSIGNES :**  Pour chacun des effets secondaires suivants, tu dois indiquer à quel point il t’a dérangé en **encerclant un chiffre** de **1 (peu dérangeant)** à **6 (très dérangeant)**. Si l’effet secondaire ne s’applique pas, encercle N/A (non-applicable). |
| --- |

|  | **Peu dérangeant** | | **dérangeant** | | **Très dérangeant** | | **N/A** |
| --- | --- | --- | --- | --- | --- | --- | --- |
| 1. La fatigue/somnolence (avoir envie de dormir plus qu’à l’habitude) | 1 | 2 | 3 | 4 | 5 | 6 | N/A |
| 2. Les nausées (avoir mal au cœur) | 1 | 2 | 3 | 4 | 5 | 6 | N/A |
| 3. Les étourdissements (avoir la tête qui tourne) | 1 | 2 | 3 | 4 | 5 | 6 | N/A |
| 4. La constipation/Douleur abdominales (avoir mal au ventre) | 1 | 2 | 3 | 4 | 5 | 6 | N/A |
| 5. Les démangeaisons (Ça pique partout, au visage, au cou, aux bras) | 1 | 2 | 3 | 4 | 5 | 6 | N/A |
| 6. Les vomissements | 1 | 2 | 3 | 4 | 5 | 6 | N/A |
| 7. Les hallucinations (voir des choses qui ne sont pas là) | 1 | 2 | 3 | 4 | 5 | 6 | N/A |
| 8. La sensation de voir double | 1 | 2 | 3 | 4 | 5 | 6 | N/A |
| 9. Les sensation bizarres désagréables (se sentir différent(e) que d’habitude | 1 | 2 | 3 | 4 | 5 | 6 | N/A |
| 10. Les sensations bizarres non-désagréables (se sentir différent(e) que d’habitude | 1 | 2 | 3 | 4 | 5 | 6 | N/A |

INTENSITÉ DE LA DOULEUR

| **CONSIGNES :**  Pour chacun des énoncés suivants, tu dois indiquer ton niveau de douleur en **encerclant un chiffre** **de 0 (absence de douleur)** à **10 (pire douleur ressentie**) sur l’échelle de douleur |
| --- |

1. Quel est ton niveau de douleur **présentement** ?

Absence de douleur

|  |  |  |  |  |  |  |  |  |  |
| --- | --- | --- | --- | --- | --- | --- | --- | --- | --- |
|  |  |  |  |  |  |  |  |  |  |

0 1 2 3 4 5 6 7 8 9 10

2. Quelle a été **ta plus grosse douleur** **dans la dernière semaine** ?

Pire douleur ressentie

Pire douleur ressentie

Pire douleur ressentie

|  |  |  |  |  |  |  |  |  |  |
| --- | --- | --- | --- | --- | --- | --- | --- | --- | --- |
|  |  |  |  |  |  |  |  |  |  |

0 1 2 3 4 5 6 7 8 9 10

3. Quel est le niveau de douleur que tu as ressenti **le plus souvent** **dans la dernière semaine** ?

Absence de douleur

Pire douleur ressentie

|  |  |  |  |  |  |  |  |  |  |
| --- | --- | --- | --- | --- | --- | --- | --- | --- | --- |
|  |  |  |  |  |  |  |  |  |  |

0 1 2 3 4 5 6 7 8 9 10

FORMAT DE LA MÉDICATION

| **CONSIGNES :**  Pour chacun des formats de médication suivants, tu dois indiquer ton degré de satisfaction en **encerclant un chiffre** de **1 (peu satisfait)** à **6 (très satisfait)**. Si le format de médication ne s’applique pas à ta situation, encercle N/A (non-applicable). |
| --- |

|  | **Peu satisfait** | | **Satisfait** | | **Très satisfait** | | **N/A** |
| --- | --- | --- | --- | --- | --- | --- | --- |
| 1. Pompe auto-analgésie (PCA) | 1 | 2 | 3 | 4 | 5 | 6 | N/A |
| 2. Médication orale (par la bouche : pilules, capsules) | 1 | 2 | 3 | 4 | 5 | 6 | N/A |
| 3. Médication appliquée avec une « patch » (collant sur le bras) | 1 | 2 | 3 | 4 | 5 | 6 | N/A |
| 4. Médication rectale | 1 | 2 | 3 | 4 | 5 | 6 | N/A |
